# Astaxanthin supplementation enhances metabolic adaptation with aerobic training in the elderly

**DOI:** 10.14814/phy2.14887

**Published:** 2021-06-10

**Authors:** Sophia Z. Liu, Ana P. Valencia, Matt P. VanDoren, Eric G. Shankland, Baback Roshanravan, Kevin E. Conley, David J. Marcinek

**Affiliations:** ^1^ Department of Radiology University of Washington Seattle WA USA; ^2^ Exercise Research Center Fred Hutchinson Cancer Research Center Seattle WA USA; ^3^ Department of Internal Medicine, Division of Nephrology University of California Davis Sacramento CA USA; ^4^ Department of Physiology & Biophysics University of Washington Seattle WA USA; ^5^ Department of Bioengineering University of Washington Seattle WA USA; ^6^ Department of Medicine University of Washington Seattle WA USA

**Keywords:** aging, anti‐oxidants, astaxanthin, fat oxidation, sex difference, training adaptation

## Abstract

Endurance training (ET) is recommended for the elderly to improve metabolic health and aerobic capacity. However, ET‐induced adaptations may be suboptimal due to oxidative stress and exaggerated inflammatory response to ET. The natural antioxidant and anti‐inflammatory dietary supplement astaxanthin (AX) has been found to increase endurance performance among young athletes, but limited investigations have focused on the elderly. We tested a formulation of AX in combination with ET in healthy older adults (65–82 years) to determine if AX improves metabolic adaptations with ET, and if AX effects are sex‐dependent. Forty‐two subjects were randomized to either placebo (PL) or AX during 3 months of ET. Specific muscle endurance was measured in ankle dorsiflexors. Whole body exercise endurance and fat oxidation (FATox) was assessed with a graded exercise test (GXT) in conjunction with indirect calorimetry. Results: ET led to improved specific muscle endurance only in the AX group (Pre 353 ± 26 vs. Post 472 ± 41 contractions), and submaximal GXT duration improved in both groups (PL 40.8 ± 9.1% and AX 41.1 ± 6.3%). The increase in FATox at lower intensity after ET was greater in AX (PL 0.23 ± 0.15 g vs. AX 0.76 ± 0.18 g) and was associated with reduced carbohydrate oxidation and increased exercise efficiency in males but not in females.

## Graphical abstract

Astaxanthin combined with endurance training promoted fat oxidation compared to training alone. Astaxanthin led to carbohydrate sparing and improved exercise efficiency especially in older males.

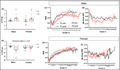

## 1 INTRODUCTION

Aging is associated with reduced cardiovascular fitness and a decline in skeletal muscle quality and quantity. Maximal oxygen consumption (VO_2max_) has been shown to decline 10% per decade after the age of 30, while a decline in VO_2max_ is directly associated with increased risk of mortality across multiple chronic conditions (Gries et al., 1985; Myers et al., 2002). The ability of skeletal muscle to serve as major metabolic reservoir to maintain energy balance and provide metabolic fuel during daily activity is also negatively impacted by aging: from reduced muscle strength and size (sarcopenia) to reduced mitochondrial capacity (ATPmax), coupling of oxidative phosphorylation (P/O ratio), elevated oxidative stress, and disruption of cell redox homeostasis (Amara et al., 2007; Chambers et al., 1985; Lexell et al., 1988; Marcinek & Siegel, 2013; Siegel et al., 2013). Therefore, protective therapeutics that can improve mitochondrial function, reduce chronic inflammation, or reduce reactive oxygen and nitrogen species (ROS/RNS), are attractive targets for clinical studies. The benefits of endurance exercise for improved cardiovascular, metabolic, and muscular health are well established (Goodpaster et al., 1985; Gries et al., 1985; Holloszy & Coyle, 1984). However, in aged individuals sustained inflammation and increased chronic oxidative stress may contribute to suboptimal results (Joseph et al., 2016; Lavin et al., 1985; Trappe et al., 2011). Therefore, interventions to optimize exercise‐induced adaptations in aging continue to be evaluated (Kidd, 2011; Labonte et al., 2008; Trappe & Liu, 1985).

Astaxanthin (AX) is a powerful carotenoid that has gained growing interest during the last decade due to its strong antioxidant capacity as well as anti‐inflammatory properties (Kidd, 2011; Yoshihara et al., 2019). Astaxanthin has been consumed as a nutritional supplement for approximately thirty years with no documented adverse events (Capelli et al., 2019). Its unique structure allows for the molecule to insert into a lipid bilayer to scavenge reactive oxygen species (ROS) and free radicals in both the inner and outer layer of the cell membrane (Kidd, 2011).

Benefits of AX supplementation on exercise performance have been reported in mice and humans. In mice, AX supplementation increased exercise capacity and fat oxidation during both swimming and treadmill exercise to exhaustion (Aoi et al., 2008; Ikeuchi et al., 2006). The increased time to exhaustion was related to increased lipid oxidation, reduced blood lactate, and reduced liver and muscle glycogen utilization (Aoi et al., 2008; Ikeuchi et al., 2006; Liu et al., 2014). The effect of AX on muscle metabolism and performance is less clear in humans, with some showing a reduction in injury markers (Djordjevic et al., 2012) but not others (Bloomer et al., 2005), or benefits in strength (Malmsten & Lignell, 2008) in healthy male athletes. Earnest et al. in 2011 reported improved 20 km time trial performance with no difference in fat or carbohydrate oxidation during sub‐max cycling in endurance trained male cyclists (Earnest et al., 2011). Res et al. observed no effect on either time trial or fat oxidization in endurance trained male athletes (Res et al., 2013). It worth noting that most AX supplementation studies have been done in highly trained male subjects that may not necessarily benefit from AX supplementation as a result of their rigorous training.

Given the potential of AX to improve muscle performance and metabolic health, we sought to test the effects of AX supplementation in older individuals. In addition, we sought to assess sex differences as most studies have been conducted on males. We previously reported that a combination of AX supplementation and interval incline walking improved muscle strength, size, and specific force in a healthy elderly population (Liu et al., 2018). Furthermore, this improvement from our aerobic‐based training program is similar to that observed with resistance training, which is the most common mode of exercise for treatment of sarcopenia (Fragala et al., 2019; Jubrias et al., 1985). The greater benefits of AX supplementation in the elderly compared to previous work published on healthy trained individuals, is consistent with previous studies testing the effects of redox‐targeted drugs on aged mice (Siegel et al., 2013) that demonstrated greater benefits in aged groups compared to young, or anti‐inflammatories in older participants (Trappe et al., 2011) that demonstrated greater benefits compared to resistance training alone.

The goal of this report is to provide insights on metabolic adaptation with combined AX and aerobic training to test whether AX enhances the metabolic benefits of exercise training in healthy older males and females. We tested the hypotheses that AX supplementation in combination with endurance training facilitates improvements in endurance and fat oxidation, and that the AX effect will be similar in both sexes.

## 2 METHODS

### 2.1 Study design

This randomized, double‐blind, placebo‐controlled study was conducted at the University of Washington Medical Center and the Fred Hutchison Cancer Research Center. Older adults between the ages of 65 and 82 were recruited through public lectures, mailers, posted advertisements, and referrals from prior studies. All participants gave written informed consent consistent with the Declaration of Helsinki in a project approved by the University of Washington and Western Institutional Review Boards, which was registered on ClinicalTrials.gov (NCT03368872). The details on recruitment process, research design, randomization, and blinding process have been reported previously (Liu et al., 2018). Briefly, participants attended up to three visits to the laboratory. At baseline (V1) participants performed a graded exercise test (GXT), tibialis anterior (TA) muscle endurance test, and had blood drawn for blood metabolic panel and AX level measurements, before being blindly randomized to the AX or PL group. After one month of supplementation alone, participants returned to the lab (V2) for a blood test and to begin endurance training (ET). Following 12 weeks of ET with continued supplementation of AX or PL, participants returned for a final visit (V3) for GXT, blood tests, and TA muscle endurance test.

### 2.2 Participants

This is a secondary analysis of a study describing the effects of AX supplementation on muscle strength (Liu et al., 2018). To be included in the study subjects had to be healthy without serious chronic conditions, be able to perform activities of daily living without assistance, and be able to speak and read English fluently. Each subject had a physical examination, resting and exercise echocardiogram, and blood testing to ensure they were healthy. Out of the 58 enrolled participants, 42 completed the TA muscle performance testing (see previous publication for details)(Liu et al., 2018), and 40 completed the graded exercise test (GXT), specifically 17 males and 23 females (one of the female had a high aerobic capacity >2SD higher than the mean and was excluded from analysis).

### 2.3 Intervention

The dietary formulation of AX consisted of astaxanthin (12 mg), tocotrienol (10 mg), and zinc (6 mg; AstaReal AX, Bellevue, WA) and was consumed as two capsules per day. The AX dose was determined based on safety studies reported previously (Kidd, 2011). Details of ET have been published previously (Liu et al., 2018). Briefly, ET consisted of a 12‐week program in which participants walked on the treadmill 3x/week. Training progressed in three steps: (i) familiarization with the treadmill protocol (weeks 1 and 2) involved 8–10 intervals at an incline between 6%–12% for ~1 min each and recovery for 2 min, (ii) baseline interval training (weeks 3–7) involved 1–1.5 min exercise in 8–10 intervals to achieve 70%–80% HRmax with 2–3 min of recovery exercise between intervals, and (iii) ramping up (weeks 8–12) involved 1.5–2 min exercise in 10–12 intervals to achieve 80%–85% HRmax with 0.5–1.0 min of recovery exercise between intervals. All exercise training was overseen by an American College of Sports Medicine certified exercise physiologist at the Fred Hutchinson Cancer Research Center.

### 2.4 Specific muscle endurance

The TA muscle strength and endurance were determined on the right leg using a custom‐built exercise apparatus. The results on muscle strength have been published (Liu et al., 2018). Skeletal muscle endurance measurement consisted of voluntary ankle dorsiflexion at 70% of maximal force with increasing contraction frequency until fatigue. Subjects voluntarily contracted the TA in response to a signal from a metronome set at 60 beats per minute and increasing 10 beats every minute. Familiarization trial was run prior to the formal test at each visit to get subjects used to the contraction and metronome. Fatigue point was defined as failure to contract in response to the metronome signal or failure to maintain 70% of maximal force.

### 2.5 Submaximal endurance test and substrate utilization

Exercise testing using standard Balke treadmill protocol (Balke & Ware, 1959) was conducted at V1 and V3. Balke protocol was chosen because walking protocol is easier to adapt for elderly participants. The test started after 5–10 mins treadmill walking familiarization. Males walked at a constant speed of 3.3 mph at increasing incline of 1% every minute. Females walked at a constant speed of 3.0 mph at increasing incline 2.5% every 3 min. Heart rate (HR), rate of perceived exertion (RPE), and blood pressure were recorded at each stage. Oxygen consumption (VO_2_) and carbon dioxide production (VCO_2_) were computed with an indirect calorimeter (MGC Diagnostics Ultima CardiO2, St. Paul, MN). The rate of sampling (gas exchange) measured breath‐by‐breath and the output values are averaged of mid 5–7 breath of every 30 seconds. All testing was terminated when the participant reached either 85% predicted HRmax or once the participant reported anRPE of 15 and had a respiratory exchange ratio (RER) ≥ 1.00.

### 2.6 Substrate utilization and exercise efficiency computations

Fat and carbohydrate (CHO) oxidation were calculated using the following equations that have been used previously with indirect calorimetry (Croci et al., 2014). Values for substrate utilization are expressed total grams integrated over the exercise period. Exercise efficiency was calculated by dividing power output (watts converted to kcal/min) by exercise energy expenditure (kcal/min) (Broskey et al., 2015). Exercise energy expenditure reflects the sum of kilocalories (kcal) derived from rates of fat and CHO oxidation (g/min multiplied by 9 and 4, respectively).

1. Respiratory Exchange Ratio (RER) = VCO_2_/VO_2_
2. Fat oxidation (g/min)=1.67VO_2_(L/min)‐1.67VCO_2_(L/min)
3. CHO oxidation (g/min)=4.55*VCO_2_ (L/min)‐3.21VO_2_ (L/min)
4. Exercise efficiency (%) = Work (kcal/min) / Energy Expenditure (kcal/min) * 100

### 2.7 Statistical analysis

To test for changes in AX blood level, total contraction, treadmill time, and RER difference in both groups we used two‐way analysis of variance (Treatment x Time) with Sidak multiple comparison test. For comparisons of the deltas between PL and AX in contraction number and RER (without the effect of sex), an unpaired, two‐tailed *t*‐test was conducted. To determine if there were differences in the deltas for fuel change and exercise efficiency between sex and supplementation, a two‐way analysis of variance (Sex X Treatment) was conducted with a Sidak's multiple comparisons test. To analyze the changes in energy expenditure, work, and total efficiency we used a mixed model analysis of variance (Sex x Time x Treatment) followed by a Sidak's multiple comparison test. Significance was assigned at α = 0.05 (*p* < 0.05). Data are reported as means ± SEM in figures and in the tables. Outliers from the data analysis were excluded if their values were more than two standard deviations from the mean, which occurred for one participant whose triglyceride level was high due to high fat diet (HFD) that was not reported before the study initiated, one participant whose TA muscle endurance was high at both at baseline and V3, and one participant with a high endurance capacity in the GXT. Statistical analyses were performed using GraphPad Prism software (San Diego, CA).

## 3 RESULTS

### 3.1 Participants

Table 1 summarizes the characteristics of the participants. Throughout the 4‐month study, only male‐PL group had a mild decrease (>1%) in body weight. All other groups remained weight stable (<1% difference).

**TABLE 1:** Subjects characteristic, lipid profile, and insulin level before (V1) and after (V3) training and supplementation (Date present as mean ± S.E.)

		PL ‐M (n = 8)	PL‐F(n = 10)	PL‐total (n = 18)	AX‐M (n = 9)	AX‐F(n = 13)	AX‐total (n = 22)
Age(yrs)		74.2 ± 1.6	70.4 ± 1.6	72.1 ± 1.3	69.2 ± 1.0	68.7 ± 0.6	69.2 ± 0.7
BW(lbs)	V1	169.1 ± 12.9	142.1 ± 5.3	154.1 ± 7.0	184.2 ± 5.0	145.4 ± 7.4	161.2 ± 6.3
	V3	166.5 ± 12.7^b^	140.7 ± 5.2	152.2 ± 6.9	185.8 ± 5.1	144.0 ± 7.1	161.1 ± 6.4
	% Change	1.5 ± 0.7	−0.9 ± 0.9	−1.2 ± 0.5	0.9 ± 0.6	−0.8 ± 0.6	−0.2 ± 0.5
Total Cholesterol (mg/dl)	V1	192.1 ± 10.9	234.3 ± 11.8	215.5 ± 9.4	211.1 ± 7.6	228.4 ± 12.7	221.3 ± 8.2
	V3	194.0 ± 9.6	229.6 ± 13.4	213.8 ± 9.4	210.2 ± 5.6	223.8 ± 13.5	218.3 ± 8.3
	% Change	1.4 ± 2.5	−2.3 ± 1.8	−0.67 ± 1.53	0.07 ± 2.32	−2.15 ± 2.62	−1.24 ± 1.79
HDL(mg/dl)	V1	66.1 ± 8.5	82.9 ± 8.5	75.4 ± 4.7	70.0 ± 4.6	70.3 ± 6.7	70.2 ± 4.3
	V3	67.3 ± 7.4	82.8 ± 5.2	75.9 ± 4.6	66.7 ± 4.8	66.3 ± 5.9	66.5 ± 8.9
	% Change	3.6 ± 3.5	−0.5 ± 2.3	1.32 ± 1.99	−4.3 ± 4.3	−4.2 ± 3.4	−4.24 ± 2.60
LDL (mg/dl)	V1	104.1 ± 5.1	134.5 ± 5.1	95.4 ± 9.4	123.3 ± 6.1	133.8 ± 11.5	96.7 ± 7.1
	V3	106.6 ± 6.0	131.2 ± 10.5	88.0 ± 8.1	123.2 ± 3.6	134.7 ± 10.4	107.5 ± 9.2
	% Change	2.8 ± 4.2	−3.0 ± 2.0	−5.5 ± 4.20	1.1 ± 3.9	2.0 ± 4.3	14.0 ± 6.0
Tri (mg/dl)	V1	109.0 ± 17.6	84.6 ± 8.8	121.0 ± 6.8	89.4 ± 8.1	102.2 ± 11.0^a^	129.5 ± 7.2
	V3	100.9 ± 13.9	77.7 ± 8.7	120.3 ± 6.9	101.2 ± 8.9	112.2 ± 14.9^a^	130.0 ± 6.3
	% Change	−4.1 ± 6.3	−6.6 ± 5.9	−0.40 ± 2.2	14.7 ± 7.2	13.5 ± 9.3	1.6 ± 2.95^d^
Insulin (µIU/ml)	V1	6.7 ± 1.4	3.7 ± 0.6	5.0 ± 0.8	6.0 ± 0.9	6.1 ± 0.8	5.7 ± 0.5
	V3	4.5 ± 1.0^b^	3.8 ± 0.5	4.1 ± 0.5	4.9 ± 0.8	4.7 ± 0.5^c^	4.7 ± 0.4
	% Change	−30.2 ± 7.2	15.2 ± 15.1	−5.0 ± 10.3	−13.2 ± 13.7	−17.9 ± 8.9	−16.0 ± 7.5

^a^ one female outlier following a high fat diet was excluded from triglyceride analysis.

^b^ *p* < 0.05 V3 compared to V1.

^c^ *p* = 0.05 V3 compared to V1.

^d^ *p* < 0.05 AX compared to PL.

### 3.2 Blood chemistry

**FIGURE 1:**
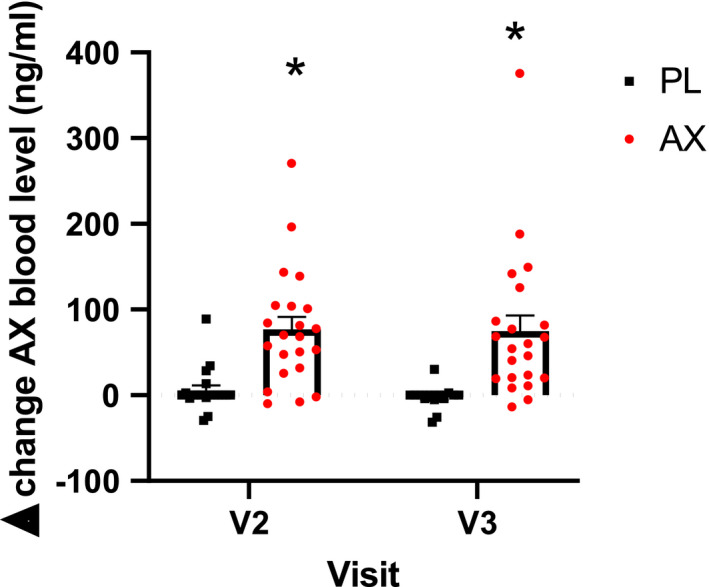
Change in serum level of AX concentration. Absolute change in serum plasma level of AX level between one month of supplementation only (V2) and post 3‐month endurance training (V3) (*p < 0.05, AX compared to PL). Data presented as mean ± S.E

Table 1 summarizes blood chemistry taken at baseline (V1), and after 3 months of ET with AX or PL supplementation (V3). Astaxanthin plasma levels were elevated after 1 month of AX or PL supplementation without ET (V2) and remained stable throughout the study (Figure 1). Changes in fasting plasma triglyceride were not different between AX and PL at V2, but significantly elevated in AX group at V3 compared to PL (although still within the healthy range of <150 mg/dL) (Table 1). In PL group, insulin level dropped after training in males (*p* < 0.05) and the drop of insulin in AX group was close to significance in females (*p* = 0.05) (Table 1). One participant following a high fat diet regiment had a blood triglyceride level that was 3 SD higher than the rest of the subjects and was excluded from the analysis. There were no differences in insulin, HDL, LDL, or total cholesterol between groups.

### 3.3 TA muscle endurance

**FIGURE 2:**
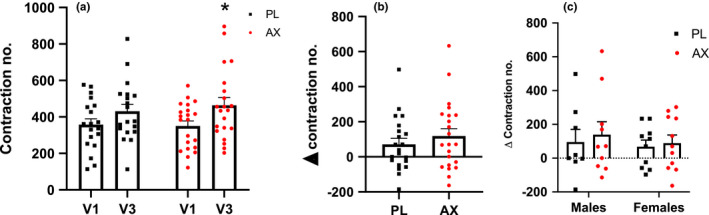
TA muscle endurance represented as the number of contractions from ankle dorsiflexion. (a): Number of contractions before (V1) and after training and supplementation (V3) (*p < 0.05, V3 compared to V1) (b): Difference in number of contractions between V3 and V1 by group. (c): Difference in number of contractions between V3 and V1 by sex. Data presented as mean ± S.E

One participant was unable to follow testing protocol and was excluded from the analysis. There was a training effect (*p* < 0.05) but no interaction effect on TA muscle endurance. Sidak's multiple comparison analysis showed this difference was driven by the AX treatment group (*p < 0.05) (Figure 2a). However, there was no treatment effect or sex difference between PL and AX in the change in TA endurance from V1‐V3 (Figure 2b,c).

### 3.4 Submaximal endurance test

**FIGURE 3:**
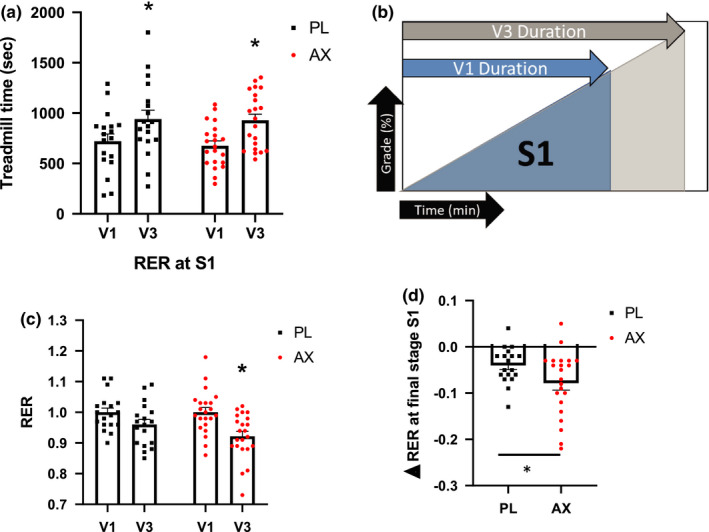
Graded treadmill exercise test. (a): Time to reach submaximal termination criteria for the GXT. (*p < 0.05, V3 compared to V1) (b): Schematics of treadmill test before (V1) and after training and supplementation (V3). (c): Respiratory exchange ratio (RER) at the last stage of S1. (*p < 0.05, V3 compared to V1) (d): Difference in RER between V3 and V1 at the end of S1. (*p < 0.05, AX compared to PL). Data presented as mean ± S.E

Endurance training increased endurance capacity in both groups leading to an approximately 4‐minute increase in the time it took for participants to meet the termination criteria for the GXT at V3 (Figure 3a). The increase in endurance capacity, the %HRmax, and the RPE were not different at the end of the exercise test between AX or PL (Supplemental Figure S1a‐b; see https://doi.org/10.6084/m9.figshare.13641338.v1). The changes in predicted VO_2max_ based on Balke treadmill equation (Pollock et al., 1976, 1982) were not different between AX and PL, but predicted VO_2max_ values increased after ET in all groups (Supplemental Figure S2; see https://doi.org/10.6084/m9.figshare.13641317.v1). In order to test the effects of the combined ET and supplementation we defined the S1 phase as the stages completed in V3 that were also completed in V1 to compare substrate utilization and exercise efficiency under the same workload (Figure 3b). Endurance training led to a decrease in the respiratory exchange ratio (RER) at the end of the S1 phase and the multiple comparison showed the reduction was primarily driven by AX group (Figure 3c), and the reduction was significantly greater in the AX group (*p* < 0.05) (Figure 3d).

### 3.5 Energy substrate

**FIGURE 4:**
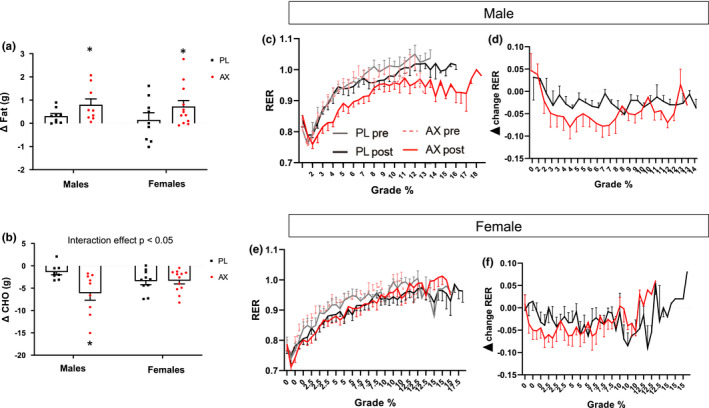
GXT metabolic fuel use and representative RER throughout different GXT stages. (a): Difference in total lipid oxidation in S1 between V3 and V1. (b): Difference in total carbohydrate oxidation in S1 between V3 and V1. (c‐f): RER as representation of fuel selection throughout different GXT stages. Data presented as mean ± S.E. (*p < 0.05, AX compared to PL)

To test for sex‐specific effects of ET and supplementation and to account for the different GXT protocols used in males and females, we analyzed both sexes separately. Total fat oxidation increased with ET in all but PL‐Female group (*p* < 0.05) (Supplemental Table S1; see https://doi.org/10.6084/m9.figshare.13641278.v1). AX further increased fat oxidation in both males and females in the S1 phase (*p* < 0.05) (Figure 4a). Carbohydrate oxidation decreased with ET in all groups and the reduction was greater in AX compared to PL for the males but not females (*p* < 0.05) (Figure 4b). The increase in relative contribution of fat to energy metabolism with AX supplementation throughout the GXT is illustrated for males and females by plotting both the absolute (Figure 4c,e) and change in RER from V1 to V3 (Figure 4d,f).

### 3.6 Exercise efficiency

**FIGURE 5:**
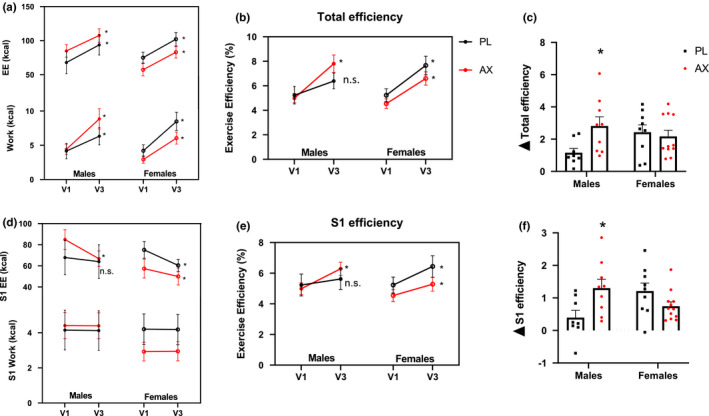
Exercise efficiency. (a): Total work and energy expenditure from GXT test. (b): Total exercise efficiency (work/ energy expenditure) (c): Difference in total efficiency between V3 and V1. (d): S1 stage work and energy expenditure from GXT test. (e): S1 efficiency between sexes. (f): Difference in S1 efficiency between V3 and V1. Data presented as mean ± S.E. (A,B,D,E *p < 0.05, V3 compared to V1; C, F *p < 0.05 AX compared to sex‐matched PL)

Participants were able to perform more work and consequently increase their energy expenditure on the GXT after ET at V3 (Figure 5a). However, total exercise efficiency increased in all groups from V1 to V3 except in males treated with PL (Figure 5b). There was an interaction effect of AX that was dependent on sex, where AX supplementation increased total exercise efficiency relative to PL in males only, (Figure 5c). Exercise efficiency can increase when the rise in work is greater relative to the rise in energy expenditure, so we compared exercise efficiency under the same workload completed at V1 (S1 phase). Females increased exercise efficiency in both AX and PL groups. The increase in exercise efficiency for males, however, was only evident in AX and not PL (Figure 5d,f).

## 4 DISCUSSION

The main findings of this report are that AX supplementation (ⅰ) increased the preference for fat oxidation under the same exercise intensity (RER ≤ 1), (ⅱ) increased exercise efficiency and reduced carbohydrate oxidation during same lower intensity exercise stages primarily in older males.

Astaxanthin is fast absorbed in the blood. A bioavailability study showed consumption of 1.25 mg AX from wild salmon (250 g) led to elevated plasma AX at day 3 and peaked by day 6 (Rufer et al., 2008). Our study showed AX levels in the blood increased after one month of supplementation and remained at the same level throughout the 3 months of exercise. Brown et al. also reported that AX supplementation for 7 days reaches significant ergogenic benefit on cycling performance (Brown et al., 2020). Despite the rapid increase in plasma AX concentration, the effect of supplementation on blood triglycerides level was only observed after 3 months of exercise training and not during the initial month of supplementation alone phase. The combined effect of AX and ET could relate to increase fat use as fuel source during exercise (Polotow et al., 2014). It is interesting to note that previous work reported that the effect of AX is positively influenced by dietary lipids through AX bioavailability studies (Kidd, 2011). The health benefit of AX, especially when combined with dietary lipids, on blood profile is supported by investigations on humans and mice (Supplemental Table S2; see https://doi.org/10.6084/m9.figshare.13641281.v1) (Choi, Kim, et al., 2011, Choi, Youn, et al., 2011; Nishida et al., 2020).

One of the main goals of exercise training interventions, especially in the elderly, is to improve exercise tolerance and metabolic health. Improvement in GXT performance after training can be due to a variety of factors beyond changes in substrate utilization, such as cardiovascular and neuromuscular adaptations. The neuromuscular adaptation from training can lead to improved functional capacity when the training and testing use the same activity mode. In this study ET led to improved treadmill time and increased predicted VO_2max_ for all groups. To distinguish metabolic adaptation from the potential that neuromuscular adaptation underlies the improved exercise tolerance we tested TA muscle endurance using an activity distinct from the training activity. The improvement in the TA endurance following ET with AX supplementation suggests a beneficial effect of AX on performance at the isolated muscle level independent of training adaptation/stimulation. This is consistent with our previous report showing improved strength with AX following ET (Liu et al., 2018). However, it is important to note that the improvements in TA endurance after ET were not different between AX and PL.

We implemented the Balke submax treadmill protocol to monitor metabolic fuel selection at various intensities of exercise. The Balke protocol is often used for populations with limited exercise capacity and the protocol varies according to sex with the male's protocol reaching higher intensities over a shorter time frame (Balke & Ware, 1959; Hagberg, 1994). We recognize this limitation and analyzed both sexes separately. To account for the likelihood that substrate utilization changes did not reach a steady‐state at each step in exercise intensity, especially in male participants where the steps were only 1 minute each, we used the sum of the substrate oxidation for all the stages in the exercise period rather than rely on the rate of substrate utilization for a given stage (Brown et al., 2020; Earnest et al., 2011; Res et al., 2013). In addition, comparing the difference within subjects also minimized the influence of non‐steady state conditions on the comparison. We found that AX supplementation increased fat utilization similarly in both males and females despite differences in protocols. The reduction in carbohydrate use after training primarily happened in male participants supplemented with AX. This sex difference could be due to the difference in exercise protocol, but could also be due to sex‐specific effect of AX. It has been documented that men utilize more carbohydrate than fat during submaximal exercise compared to women, that may be attributed to lower intramyocellular lipid and adipocyte sensitivity to lipolysis (Tarnopolsky, 2008). Thus, promoting fat oxidation with AX in males may promote a greater carbohydrate‐sparing effect than in women, as males’ initial reliance on carbohydrate was greater. The limited studies including female participants makes it even more interesting to investigate the potential sex differences in response to AX supplementation (Supplemental Table S2).

Our findings from the elderly participants improving fat oxidation from AX supplementation agree with the findings on younger individuals. For instance, a recently published performance focused study on male cyclists using a cross‐over design also reported improved performance and fat oxidation during a 40 km cycling time trail (Brown et al., 2020). Both the dynamic protocol in this study and the cross‐over study design cited above allowed testing of fat oxidation across a range of exercise intensities and accounted for individual differences on substrate utilization and exercise capacity. In contrast, two other performance focused study set the cycling intensity at 50% Watt‐max did not detect the benefits of AX on fat oxidation throughout the sub‐max cycling activity (Earnest et al., 2011; Res et al., 2013). The dynamic versus steady state (50% Watt‐max) (Earnest et al., 2011; Res et al., 2013) protocol may have partially contributed to inconsistent finding on fat oxidation in human (Supplemental Table S2).

An important variable to consider when assessing the adaptation to training or the effect of supplementation is the starting fitness level of the subjects. Our healthy elderly predicted VO_2max_ values were between ~20–30 ml/kg/min compared to 50–60 ml/kg/min from other studies of AX that recruited highly trained athletes (Earnest et al., 2011; Res et al., 2013). Fat oxidation in highly trained athletes is likely already optimized through years of training. To the best of our knowledge, only one other study has explored the effects of AX supplementation in the context of aging. Park et al (Park et al., 2013) demonstrated restored redox balance, improved mitochondrial mass, and ATP production by peripheral blood mononuclear cells of both young and geriatric dogs. In addition, AX suppressed plasma DNA oxidative damage only in geriatric dogs. Future investigations of the impact of AX supplementation in the elderly are necessary as the benefit to this population may be greater than what has been documented in younger individuals.

Older sedentary adults tend to have low levels of exercise efficiency (Broskey et al., 2015; Woo et al., 2006) that can prevent them from accomplishing daily activities and reduce their quality of life. Increasing exercise efficiency means that it would require less energy to perform a given amount of work. Similar to previous studies (Myers et al., 2002; Woo et al., 2006) we found that endurance training increased exercise efficiency in older adults regardless of sex. Interestingly, we also found that males who were supplemented with AX were more responsive than their placebo‐treated counterparts to training‐induced increases in exercise efficiency. Factors contributing to increased efficiency include improved muscle oxidative capacity, muscle capillarization, cardiac function, and mitochondrial function (Broskey et al., 2015; Conley et al., 2013; Masuki et al., 1985; Woo et al., 2006). It has been shown that supplementation with 5‐aminolevulinic acid and iron, which are involved in heme biosynthesis and can increase complex IV activity and increase ATP synthesis rate in mouse muscle (Ogura et al., 2011), promoted increases in exercise efficiency in older females participating in a home‐based walking training program (Masuki et al., 1985). Thus, AX could promote a similar mechanism as it has been shown to increase gene expression of mitochondrial complexes and ATP production (Nishida et al., 2020).

It is important to point out some potential pathways published in the literature for future clinical trials. (Aoi et al., 2008) reported that AX improved fat oxidation in mice through increased co‐immunoprecipitation of fatty acyl transferase (FAT/CD36) with carnitine palmitoyltransferase I (CPTI), and AX reduced oxidative stress‐induced modification of CPTI by hexanoyl‐lysine adduct (HEL). In contrast, other antioxidant, such as vitamin C and E, have mixed results: either enhancing or interfering with exercise adaptation (Gomez‐Cabrera et al., 2008; Peternelj & Coombes, 2011).

In summary, AX is natural antioxidant and anti‐inflammatory supplement that may have the potential to benefit muscle function and exercise tolerance in the aged population. Here we have demonstrated that combining AX and exercise training leads to improved fat oxidation, CHO sparing, and increased exercise efficiency in aged healthy subjects, especially in males. These metabolic improvements combined with the benefits of the combined intervention on improvements in muscle strength, size, and specific force indicate that incorporation of AX into an exercise training program in the elderly could enhance exercise tolerance and quality of life. While these results are intriguing, the mechanisms underlying the enhanced adaptation to exercise and sex‐specific effects on fuel selection and exercise efficiency observed in this study are still unknown. Studies combining exercise training, functional testing, and adaptive signaling will be necessary to better understand the mechanisms by which AX enhances the adaptive response to training in aged subjects.

## 5 LIMITATIONS

Data from this study demonstrated that AX improved fat utilization and reduced carbohydrate use during graded exercise especially at lower intensities (RER<1). However, this study was not designed to collect information on the biochemical mechanisms/pathways underlying these effects, which is one of our study limitations. Our subjects were instructed to limit antioxidant supplements before enrolling in the study, but lack of dietary information that may potentially influence our results.

## CONFLICT OF INTEREST

S.Z.L., A.P. V., M.D.C., K.K., E. G.S., B.R., and D.J.M. declares that there is no conflict of interest. K.E.C. has received research funds and was a Scientific Advisor for Astavita, Inc. before his death in 2019.

## AUTHOR CONTRIBUTIONS

Sophia Z. Liu, David J. Marcinek, Kevin E. Conley, and Astavita designed the research; Kevin E. Conley, Sophia Z. Liu, Matt P. VanDoren, and Eric G. Shankland performed the research; Baback Roshanravan provided clinical oversight; Sophia Z. Liu, David J. Marcinek, and Ana P. Valencia analyzed data; and Sophia Liu, Ana Valencia, and David J. Marcinek wrote the paper. As first, second, and corresponding authors, Sophia Z. Liu, Ana Valencia, and David J. Marcinek contributed equally to the study. Kevin E. Conley contributed to the manuscript, but died prior to the manuscript's submission for publication.

## Supporting information



Figures S1‐2

Table S1

Table S2
