# Supplementary figures and images for: Astaxanthin supplementation enhances metabolic adaptation with aerobic training in the elderly
Source: Physiol Rep. 2021 Jun 10;9(11):e14887. doi: 10.14814/phy2.14887 (PMC8191397; doi:10.14814/phy2.14887)

Figure S1

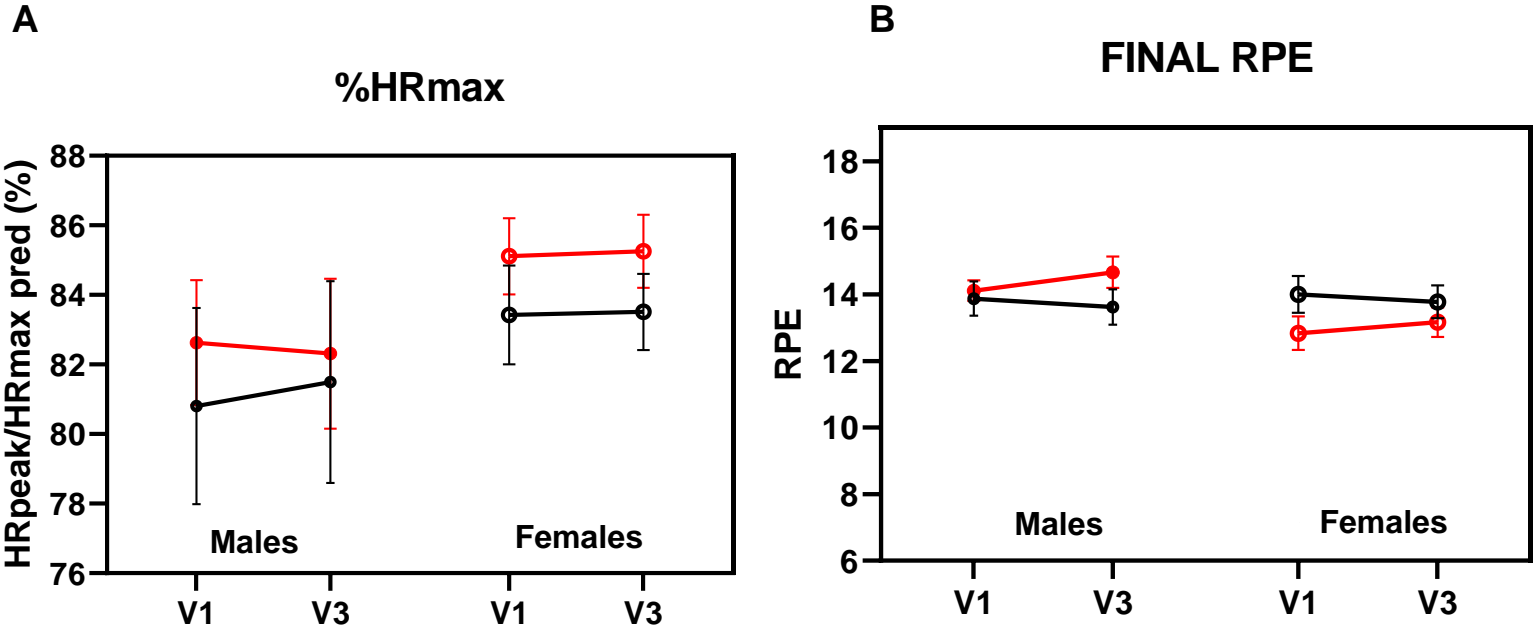

HRmax equation for age > 40yrs =  $208 - (0.7 \times \text{age})$

Figure S2

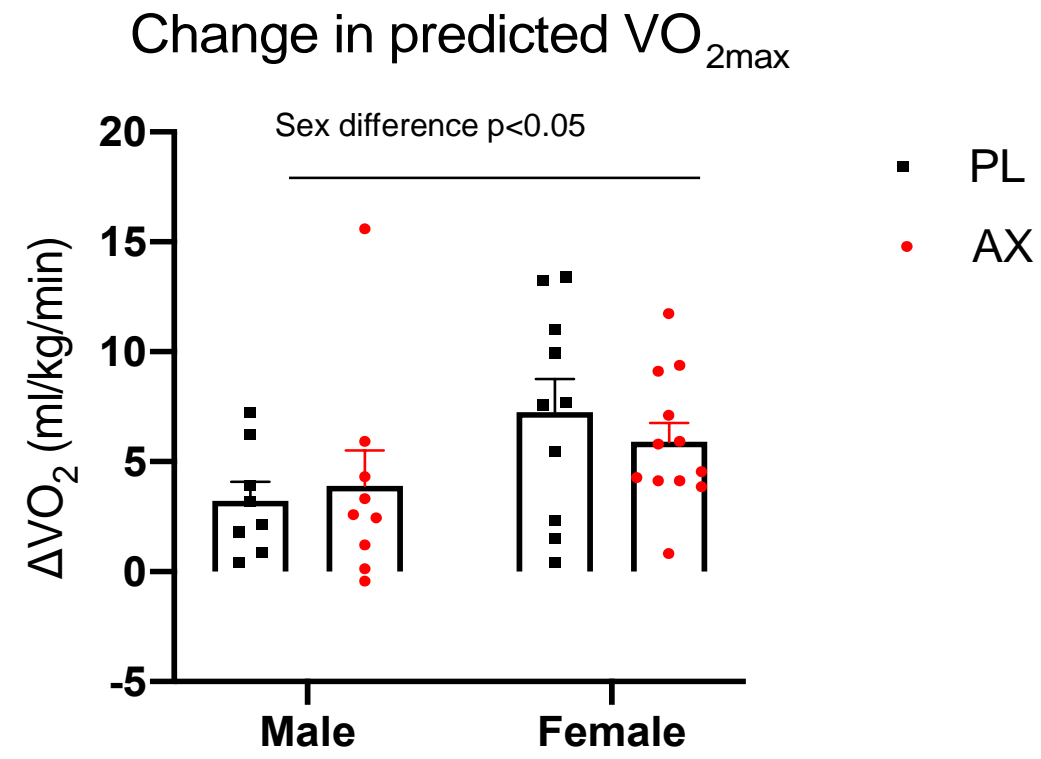

Supplement: Supplementary file 1 — Figures S1‐2 [file PHY2-9-e14887-s003.pdf]
