# Supplementary material for: Astaxanthin supplementation enhances metabolic adaptation with aerobic training in the elderly
Source: Physiol Rep. 2021 Jun 10;9(11):e14887. doi: 10.14814/phy2.14887 (PMC8191397; doi:10.14814/phy2.14887)
Supplement: Supplementary file 2 — Table S1 [file PHY2-9-e14887-s002.docx]

Table S1 Total fat oxidation before and after training (Date present as mean±S.E.)

|  | PL-Male  (n=8) | AX-Male  (n=9) | PL-Female  (n=10) | AX-Female  (n=12^#^) |
| --- | --- | --- | --- | --- |
| V1 | 1.38±0.2 | 2.46 ±0.5 | 2.48 ±0.4 | 2.11 ±0.3 |
| V3 | 1.76±0.3* | 3.51±0.5* | 3.26 ±0.5 | 3.44 ±0.4* |

- p<0.05 simple paired t-test comparing V3 to V1
- # one out layer excluded from AX-women based on high aerobic capacity didn’t fit for the Balke exercise protocol
