# Supplementary material for: Astaxanthin supplementation enhances metabolic adaptation with aerobic training in the elderly
Source: Physiol Rep. 2021 Jun 10;9(11):e14887. doi: 10.14814/phy2.14887 (PMC8191397; doi:10.14814/phy2.14887)
Supplement: Supplementary file 3 — Table S2 [file PHY2-9-e14887-s001.docx]

**Table S2 AX supplementation human study literature review**

| **Author, year** | **Subject** | **Does** | **METHODS** | **Key finding** |
| --- | --- | --- | --- | --- |
| Sawaki et. al. 2004 (11) | college student | 4wks | fasting 1200M time trail | Lactic acid lower 2mins post 1200m run (not 4,8,10min post) |
| Earnest et. al. 2011(7) | endurance trained Male (18-39) | 4mg/d for 4wks | overnight fasting+ 2hr submax followed by 20k TT | No difference in blood chemistry (cholesterol, triglyceride) changes, fat or CHO oxidation during sub-max cycling; AX improved TT performance compares to PL |
| Res. et. al. 2012(10) | Highly trained Male triathlete/cyclist | 20mg AX (+10mg VE) /Day for 4wks | Overnight fasting+ 1hr 50%Wattmax +1hrTT | No improvement on TT or Fat oxidation during 50%Wmax (RER calculation)  No changes on MDA (anti-oxidant marker), insulin, lactate, or fatty acid throughout TT |
| Choi et. al. 2011(5) | Sedentary/obese adult | 20mg for 12 wks | lipide profile and prooxidative stress marker | lower LDL in AX group at 12wk  decrease MDA, @ 4,8 and 12 wk |
| Choi et. al. 2011(4) | Overweight young adult compares to normal BW control group | low 5mg and high 20mg group for 3 weeks | blood analysis of lipide profile and oxidative stress marker at baseline & 3wks | AX blood level higher in 20mgMDA. ISP (OS marker) lower compare to baseline for both dose  No dose group difference on antioxidant or OS marker |
| Djordjevic et. al. 2012(6) | Young male soccer player | 4mg/d for 90days (3 months) | 90 days training + 2hr soccer exercise; blood sample before & after supplementation and after acute 2hr exercise | post ex CK and AST lower in AX group vs PL.; training increased O2- level in both group, O2 - increased after acute exercise only in PL ; AX+ training could prevent exercise induced radical production and depletion of nonenzymatic antioxidant defense |
| Baralic et. al. 2015(1) | young male soccer player | 4mg/d for 90 days | 90day Soccer training blood and saliva collection before and after supplementation & training | Exercise training alone reduced muscle damage CK and LDH from baseline, AX greater reduced the inflammatory markers restore redox balance preventing inflammation by rigorous training  AX reduced the training induced increase in inflammatory markers (SCP and total leukocyte vs. PL) |
| Liu et. al. 2018(8) | >65 elderly healthy | 4month (12mg AX +10mg Vt E) | 4month supplementation+3month TM interval training | increased strength, size and specific force similar to resistance training |
| Bloomer et. al. 2005(2) | Resistance trained young men | 3 wks 4mg/d | 10 sets of 10 repetitions eccentric at 85% of ERM; muscle soreness CK and performance before and through 96 hr post RE | No change in CK LDH and muscle soreness or exercise performance; greater decrease in MDF (dynamic force) 10-72 hr post ex in AX group vs. PL |
| Malmsten et. al. 2008(9) | male student | 4mg/d for 6 months | @3- & 6-month Wingate test squad test and fitness test | no changes in Wingate performance, increase number of squadding endurance strength in AX group after 6-month compare to PL |
| Brown et. al. 2020(3) | Male cyclist (VO2 ~56.5 ± 5.5 mL/kg/min) | 12mg/day for 7 days | Cross over design  14 days washout  40km cycling time trail | Time to complete 40km reduced 1.2± 1.7% with AX  FATox improved and RER lowered between 39-40km. |

**Reference**

1. **Baralic I, Andjelkovic M, Djordjevic B, Dikic N, Radivojevic N, Suzin-Zivkovic V, Radojevic-Skodric S, and Pejic S**. Effect of Astaxanthin Supplementation on Salivary IgA, Oxidative Stress, and Inflammation in Young Soccer Players. *Evid Based Complement Alternat Med* 2015: 783761, 2015.

2. **Bloomer RJ, Fry A, Schilling B, Chiu L, Hori N, and Weiss L**. Astaxanthin supplementation does not attenuate muscle injury following eccentric exercise in resistance-trained men. *Int J Sport Nutr Exerc Metab* 15: 401-412, 2005.

3. **Brown DR, Warner AR, Deb SK, Gough LA, Sparks SA, and McNaughton LR**. The effect of astaxanthin supplementation on performance and fat oxidation during a 40 km cycling time trial. *J Sci Med Sport* 2020.

4. **Choi HD, Kim JH, Chang MJ, Kyu-Youn Y, and Shin WG**. Effects of astaxanthin on oxidative stress in overweight and obese adults. *Phytother Res* 25: 1813-1818, 2011.

5. **Choi HD, Youn YK, and Shin WG**. Positive effects of astaxanthin on lipid profiles and oxidative stress in overweight subjects. *Plant Foods Hum Nutr* 66: 363-369, 2011.

6. **Djordjevic B, Baralic I, Kotur-Stevuljevic J, Stefanovic A, Ivanisevic J, Radivojevic N, Andjelkovic M, and Dikic N**. Effect of astaxanthin supplementation on muscle damage and oxidative stress markers in elite young soccer players. *J Sports Med Phys Fitness* 52: 382-392, 2012.

7. **Earnest CP, Lupo M, White KM, and Church TS**. Effect of astaxanthin on cycling time trial performance. *Int J Sports Med* 32: 882-888, 2011.

8. **Liu SZ, Ali AS, Campbell MD, Kilroy K, Shankland EG, Roshanravan B, Marcinek DJ, and Conley KE**. Building strength, endurance, and mobility using an astaxanthin formulation with functional training in elderly. *J Cachexia Sarcopenia Muscle* 9: 826-833, 2018.

9. **Malmsten CL, and Lignell Å**. Dietary Supplementation with Astaxanthin-Rich Algal Meal Improves Strength Endurance

– A Double Blind Placebo Controlled Study on Male Students-. *Carotenoid Science* 13: 2008.

10. **Res PT, Cermak NM, Stinkens R, Tollakson TJ, Haenen GR, Bast A, and Van Loon LJ**. Astaxanthin supplementation does not augment fat use or improve endurance performance. *Med Sci Sports Exerc* 45: 1158-1165, 2013.

11. **Sawaki K, Yoshigi H, Aoki K, Koikawa N, Azumane A, Kaneko K, and Yamaguchi M**. Sports Performance Benefits from Taking Natural Astaxaxxthin * Characterized by Visual Acuity and Muscular Fatigue Improvement in Humans. 2004.
